# Type 2 diabetes originated from non-alcoholic fatty liver disease

**DOI:** 10.1093/lifemeta/load007

**Published:** 2023-02-21

**Authors:** Xuelian Xiong, Xiaoying Li

**Affiliations:** Department of Endocrinology and Metabolism, Zhongshan Hospital, Fudan University, Shanghai 200032, China; Department of Endocrinology and Metabolism, Zhongshan Hospital, Fudan University, Shanghai 200032, China

**Keywords:** non-alcoholic fatty liver disease, gluconeogenesis, hepatokine, exercise

## Abstract

Both non-alcoholic fatty liver disease (NAFLD) and type 2 diabetes mellitus (T2DM) are highly prevalent metabolic liver diseases. Accumulating epidemiological evidence now indicates that NAFLD and T2DM are strongly associated, yet the causative relationship remains to be elucidated. Liver serves as a hub for nutrient and energy metabolism in the body. Here we demonstrated the pathogenesis linking NAFLD to T2DM through a series of studies and the attenuation of T2DM progression after NALFD improvement in cohort study. We proposed the urgent necessity of NAFLD management and NAFLD drug development, which might be novel therapeutic avenues for T2DM.

Type 2 diabetes mellitus (T2DM) and non-alcoholic fatty liver disease (NAFLD) have become a global epidemic with rapidly increasing prevalence and incidence [1]. Epidemiological studies have established that T2DM and NAFLD are strongly associated, yet the mechanisms underlying the potential causative relationship between these conditions remain unclear.

## Epidemiological characteristics of NAFLD and T2DM

NAFLD is the most prevalent chronic liver disease worldwide. It is estimated that NAFLD affects one-third of the general population, and up to 50% overweight and obese people [2]. Over the past decades, NAFLD has been rising in parallel with the prevalence of obesity and T2DM. The prevalence of diabetes among adults is around 10% and much lower than that of NAFLD, which implies that NAFLD occurs prior to diabetes and is associated with diabetes development [3]. A number of cohort studies demonstrate that NAFLD is a risk factor for incident T2DM. Individuals with NALFD have a 2- to 5-fold increase in developing T2DM than those without NAFLD [4]. This increase in risk for T2DM by NAFLD remains after adjustment for body weight, physical activity, family history, and other established metabolic risk factors [5]. Of note, the attenuation or aggravation of NAFLD is also associated with a reduction or an increase in the risk of T2DM [6]. Meanwhile, co-existence of diabetes makes NAFLD patients more likely to progress to severe stages including NASH, liver fibrosis, and end-stage liver disease [7, 8]. NAFLD and diabetes act synergistically and lead to a more severe adverse outcome than either condition alone.

## Pathogenesis linking NAFLD to T2DM

Obesity and insulin resistance are key pathogenic factors involved in the development of T2DM. These two pathological conditions are also frequently observed in NAFLD. In the past several decades, our group has been working on elucidating the complex link between NALFD and T2DM. NAFLD is characterized by the over accumulation of triglycerides in the liver. The increase of intrahepatic triglyceride contents can be triggered either way: (i) an increase of lipid synthesis including *de novo* lipogenesis and hepatic uptake of free fatty acids released from adipose tissues, and (ii) a decrease of hepatic triglyceride clearance including fatty acid β-oxidation and very low-density lipoprotein export [9]. The increase of *de novo* lipogenesis makes the major contribution to NAFLD development. Sterol regulatory element-binding protein 1c (SREBP-1c) plays a key role in regulating lipogenesis by upregulation of fatty acid synthase, stearoyl-CoA desaturase-1, and acetyl-CoA carboxylase 1/2 gene expressions. SREBP-1c is activated by overnutrition, obesity, inflammation, oxidative stress, endoplasmic reticulum (ER) stress, etc. Our findings demonstrated that farnesoid X receptor (FXR), a member of the nuclear receptor superfamily, could negatively regulate *de novo* lipogenesis by counteraction against SREBP-1c. FXR expression is markedly reduced in high-fat diet and genetically obese (*ob*/*ob*) mice. Deletion of hepatic FXR leads to hepatosteatosis and in contrast, overexpression of FXR in the liver of obese mice greatly ameliorates hepatosteatosis. Our study revealed that Yin Yang 1 suppresses the transcriptional expression of FXR in obese mice [10]. The dysfunction of FXR is also involved in NAFLD development in elderly people and postmenopausal women [11, 12]. Thus, FXR deficiency plays a causal role in the development of NAFLD, and FXR agonist, obeticholic acid, would be a very promising drug against NAFLD.

For years, the potential effect of low-grade inflammation on insulin resistance and T2DM has been investigated [13]. Pre-inflammatory cytokines produced by macrophage in the liver interrupt insulin signaling in the liver, as well as in adipose tissue and skeletal muscle. Hepatic insulin resistance is presented in most NAFLD patient, which results in unsuppressed hepatic glucose production (HGP) and increased blood glucose level.

Insulin resistance of peripheral tissues and pancreatic beta cell dysfunction have long been considered as two major pathophysiological processes of T2DM. The liver is a central player in maintaining glucose homeostasis through regulation of HGP or hepatic glucose utilization depending on blood glucose level. Excessive gluconeogenesis and hepatic glucose output result in fasting hyperglycemia, which is characterized by T2DM. Our previous work revealed that hepatic 17-hydroxyprogesterone (17-OHP) concentrations were increased in NAFLD due to aberrant expression of cytochrome P450 17A1 (CYP17A1). 17-OHP is an intermediate of cortisol biosynthesis, which augments gluconeogenesis in a glucocorticoid receptor-dependent manner and increases glucose production by the liver. Selective inhibitor targeting Cyp17A elicited robust glucose-lowering effect in diabetic mouse models [14]. We also found a chronic low-grade ER stress in the liver of obese NAFLD mice. The sustained ER stress promoted gluconeogenesis through induction of ubiquitin-specific peptidase 14 and therefore stabilization of 3ʹ,5ʹ-cyclic monophosphate-responsive element-binding protein [15]. Together, NAFLD appears to enhance gluconeogenesis through several different mechanisms.

Beyond cell-autonomous mechanisms, several hepatokines have been shown to regulate insulin sensitivity and glucose homeostasis, including fetuin-A. Fetuin-A acts as an endogenous ligand of Toll-like receptor 4 to promote inflammation and insulin resistance in adipose tissue [16]. We found that fetuin-A could be ubiquitinated by F-box and WD repeat domain containing 7 (FBXW7), and subsequently degraded by the proteasomal pathway. Downregulation of hepatic FBXW7 in NAFLD results in accumulation of fetuin-A and systemic insulin resistance [17]. Periostin, another hepatokine that is upregulated in NAFLD, was sufficient to promote hepatic insulin resistance through its activation of c-Jun N-terminal kinase (JNK)/c-Jun signaling pathway and subsequent suppression of fatty acid oxidation [18].

## Therapeutic avenues for T2DM targeting hepatic steatosis

Accumulating evidence indicates that NAFLD may precede and exacerbate the development of T2DM. We hypothesized that improvement of NAFLD may reduce the risk of incident T2DM. Currently, there is no approved drugs for the treatment of NAFLD, and lifestyle modification is still the most effective therapy for NAFLD. In a randomized clinical trial conducted by our group, we demonstrated that exercise significantly reduced hepatic lipid content and moderate exercise was equally effective in improving NAFLD as vigorous exercise [19]. The participants were followed up for 10 years to elucidate the incidence of T2DM. Excitedly, we found a 50% reduction of T2DM incidence in both moderate and vigorous exercise groups as compared with the non-exercise control group [20]. In addition, the improvement of hepatic triglyceride content during 1-year exercise intervention is an independent factor for the reduction of the risk for T2DM after adjustment for metabolic confounding factors. Calorie-restricted diet is known to be able to significantly improve NAFLD and also be very effective in delaying the onset of diabetes [21, 22]. Several hypoglycemic agents, including metformin, glucagon-like peptide-1 receptor agonists, and sodium-glucose cotransporter 2 inhibitors, have been shown to exert beneficial effects on NAFLD [23]. Yet more studies are needed to address whether the glucose-lowering effect of these anti-diabetic drugs was partially mediated by the remission of NAFLD.

## Future perspectives

**Figure 1:**
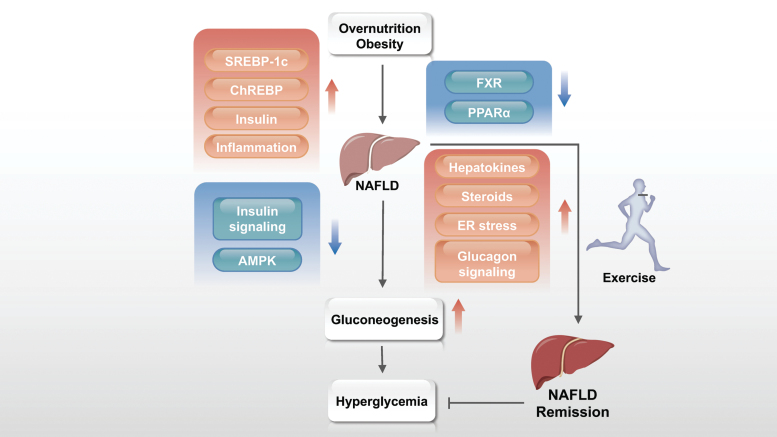
Hyperglycemia originated from NAFLD. The figure illustrates the pathogenesis of hepatic steatosis and eventually hyperglycemia under overnutrition and obesity. Exercise could effectively improve NAFLD and reduce the incidence of T2DM over the long term.

We believe that the pathophysiological connections between NAFLD and T2DM are much more intricated than currently appreciated (Fig. 1). Further research is warranted to fully establish the causal relationship between NAFLD and T2DM. The studies discussed above underscore the necessity for screening, diagnosis, and management of NAFLD. The idea that improvement of NAFLD attenuates T2DM progression might highlight the urgency of NALFD drug development.
